# Effects of acute voluntary loaded wheel running on BDNF expression in the rat hippocampus

**DOI:** 10.20463/jenb.2017.0034

**Published:** 2017-12-31

**Authors:** Minchul Lee, Hideaki Soya

**Affiliations:** 1. Department of Sports Medicine, College of Health Science, CHA University, Pocheon Republic of Korea; 2. Laboratory of Exercise Biochemistry and Neuroendocrinology, Faculty of Health and Sport Sciences, University of Tsukuba, Ibaraki Japan

**Keywords:** voluntary loaded wheel running, hippocampus, brain-derived neurotrophic factor (BDNF), work level

## Abstract

**[Purpose]:**

Voluntary loaded wheel running involves the use of a load during a voluntary running activity. A muscle-strength or power-type activity performed at a relatively high intensity and a short duration may cause fewer apparent metabolic adaptations but may still elicit muscle fiber hypertrophy. This study aimed to determine the effects of acute voluntary wheel running with an additional load on brain-derived neurotrophic factor (BDNF) expression in the rat hippocampus.

**[Methods]:**

Ten-week old male Wistar rats were assigned randomly to a (1) sedentary (Control) group; (2) voluntary exercise with no load (No-load) group; or (3) voluntary exercise with an additional load (Load) group for 1-week (acute period). The expression of BDNF genes was quantified by real-time PCR.

**[Results]:**

The average distance levels were not significantly different in the No-load and Load groups. However, the average work levels significantly increased in the Load group. The relative soleus weights were greater in the No-load group. Furthermore, loaded wheel running up-regulated the BDNF mRNA level compared with that in the Control group. The BDNF mRNA levels showed a positive correlation with workload levels (r=0.75), suggesting that the availability of multiple workload levels contributes to the BDNF-related benefits of loaded wheel running noted in this study.

**[Conclusion]:**

This novel approach yielded the first set of findings showing that acute voluntary loaded wheel running, which causes muscular adaptation, enhanced BDNF expression, suggesting a possible role of high-intensity short-term exercise in hippocampal BDNF activity.

## INTRODUCTION

It is well known that several weeks of physical exercise affect not only the body, but also brain and mental functions. Previous studies have indicated that exercise can improve cognitive function, reduce anxiety and depression ^1^, and protect the brain from neurodegenerative disorders ^2^. In particular, physical exercise enhances synaptic plasticity in the hippocampus, a site critical for neurogenesis and cognitive function ^3^. 

Brain-derived neurotrophic factor (BDNF), a growth factor of the neurotrophin family, is a key protein supporting the growth, development, and survival of neurons ^4^^-^^6^. Accumulating evidence also suggests that BDNF plays an important role in cognitive function ^7^ and synaptic plasticity ^8^. Previous studies have already shown that physical exercise increases the levels of BDNF mRNA and protein ^9^^,^ ^10^ and its high-affinity receptor tropomyosin-related kinase B (TrkB) ^11^^-^^13^ in the hippocampus. Alterations in BDNF are apparently necessary for the effects of exercise on brain plasticity to manifest in rodents, as blocking BDNF signaling inhibits the improvements in learning and memory following exercise training ^7^. The effects of exercise on hippocampal plasticity are similar to those of BDNF application, suggesting that exercise-induced changes in BDNF are important for the exercise-enhanced hippocampal functions. 

The exercise condition (intensity, duration, etc.) that best generates beneficial effects are still a matter of debate. The type of exercise may be particularly important. Voluntary wheel running is an intermittent mode of exercise, and usually only the running distance can be evaluated. Indeed, the running distance has been found to positively correlate with BDNF mRNA levels ^9^. In contrast, loaded wheel running (LWR) allows for the application of a specific load on a voluntary running wheel and is a useful exercise model to increase work levels in the form of increased energy expenditure without introducing physical and psychological stressors (such as electrical shock, weight vest, food deprivation, duration, etc.). LWR can elicit fast-twitch muscle hypertrophy and an increase in oxidative capacity (citrate synthase [CS] activity), probably due to the relatively high intensity and short term of the exercise ^14^^-^^16^. Therefore, whether LWR reproduces the beneficial effects of normal wheel exercise on hippocampal adaptation remains unclear. 

Our previous studies have already shown that the brain responds to continued running in intensity-dependent patterns. We examined hippocampal neurogenesis and cognitive functions in the adult rat hippocampus after 4 weeks of voluntary wheel running with and without a load ^17^^-^^19^. We found that the average work levels significantly increased and the average running distance decreased to about half in the voluntary resistance exercise with 30% of body weight group compared to those in the free wheel running without a load group. This in turn elicited muscular adaptation for the fast-twitch plantaris muscle without causing any negative stress effects. Furthermore, both resistance wheel running and free wheel running improved cognitive function and increased hippocampal BDNF signaling. 

Therefore, we further studied these phenomena by investigating whether 1 week of acute voluntary wheel running with additional load can change the positive effects on BDNF gene expression and signaling in the hippocampus. 

## METHODS

### Animals

Ten-week-old male Wistar rats (300–320 g; SEAS, Co., Ltd., Saitama, Japan) were randomly allocated to three groups: ^1^ housed in standard cages and used as non-active controls (Sed group); ^2^ housed in cages with conventional free running wheels with a constant base resistance of 4.5 g (No-load group), and ^3^ housed in cages with resistance running wheels with an adjustable resistance (Load group). The rats of each group were designated for assessments of both muscular adaptation and hippocampal BDNF signaling (n = 8/group). All rats were individually housed and kept in a controlled environment with a 12 h–12 h light–dark cycle (lights on at 8:00 a.m.) and given ad libitum access to food and water. All the experiments were performed in accordance with protocols approved by the University of Tsukuba Animal Experiment Committee, based on the NIH Guidelines for the Care and Use of Laboratory Animals (NIH publication, 1996). 

### Running wheel and exercise protocols

Both running wheel groups were housed individually and had free access to a specially designed running wheel apparatus (diameter = 31.8 cm, width = 10 cm; Rat Analyzer KI-103, Aptec Inc., Kyoto, Japan). The resistance attached to the wheel could be changed arbitrarily within a range of 0 to 200 g. The resistance necessary to overcome the inertia of the wheel at its minimum load was 4.5 g. This load apparatus and its protocols for the loaded running wheel were similar to those previously described ^14^. 

In the present study, the rats were allowed to run voluntarily in the wheel 24 h/day. The rats in the Load group exercised with 30% of body weight for the first week. The choice of 30% of body weight as a resistance was based upon the findings of Kasuga et al [20], with the same apparatus as above ^14^. They reported that 30% of body weight for resistance wheel running is sufficient to enhance muscular adaptation. 

Daily work levels (J) as total energy expenditure during exercise were calculated and expressed relative to body weight and day as follows: Work = Force (N) x Distance (m) / body weight (kg) / day, where force is the resistance of the wheel and distance is the number of revolutions times the circumference of the wheel. 

### Sample collection

Between 08:00 and 12:00, after completion of the exercise test, the animals were immediately decapitated using a guillotine. To measure stress levels, the adrenals and thymus were excised and weighed, and to measure muscle adaptation, the soleus and plantaris muscles were excised and weighed, then stored at -80°C before use. For the mRNA analysis, the hippocampi were rapidly dissected, snap-frozen in liquid nitrogen, and stored at -80°C before use. 

### Measurement of skeletal muscle CS activity

The CS activity in the plantaris and soleus muscles was measured using a method described previously ^21^. The CS activity in muscles was determined using the Citrate Synthase Assay Kit (Sigma, Saint Louis, Missouri, USA) following the manufacturer’s instructions. 

### Isolation of RNA and quantitative real-time PCR

Total RNA was extracted by the method described previously ^22^, and RNA concentration was determined by spectrophotometry at 260 nm. cDNA synthesis was carried out using murine leukemia virus reverse transcriptase (Applied Biosystems, Foster City, CA) and random hexamer primer sets (Takara Shuzo, Shiga, Japan) according to the manufacturer's protocol. Gene expression levels of total BDNF and related molecules were determined by quantitative real-time PCR (PE-ABI Prism 7300, Applied Biosystems, Foster City, CA) using a fast start universal SYBR green master mix (Roche Applied Science, Mannheim, Germany) according to the protocol provided by the manufacturer. Primer 3 software ^23^ was used to design reaction primers and the sequences were as follows: BDNF forward: 5’-gcggcagataaaaagactgc-3’, BDNF reverse: 5’-gccagccaattctctttttg-3’, TrkB forward: 5’-gacctgatcctgacgggtaa-3’, TrkB reverse: 5’-tggtcacagacttcccttcc-3’, β-actin forward: 5’-aaccctaaggccaaccgtga-3’, β-actin reverse: 5’-cagggacaacacagcctgga-3’. In brief, after 10 min of incubation at 95°C, PCR was carried out over 40 cycles at 95°C for 15 s, 60°C for 30 s and 72°C for 30 s. As a validated endogenous control, β-Actin was amplified in a separate reaction for normalization. Each sample was processed in duplicate and melting curve analysis was performed on all samples. 

### Statistical analysis

Data are expressed as mean ± SEM. The statistical significance between different groups was assessed using one-way ANOVA followed by Fisher’s PLSD post hoc test or Student’s t-test whenever appropriate. Pearson’s correlation analysis was used to determine the relationship between BDNF mRNA and work levels. Statistical significance was established at P < 0.05. 

## RESULTS

### Effect of LWR on body weight and exercise performance

During and at the end of the study, the average body weight was lower in both No-load and Load exercise groups compared to that in the Sed group (P < 0.05; Table 1). There was no significant change in the average running distance, time, and velocity (Table 2). In contrast, average work levels significantly increased by about 23.6-fold in the Load group (670.4 N·m/kg BW/day) compared to those in the No-load group (28.4 N·m/kg BW/day) (P < 0.001; Table. 2). 

**Table 1:** Effects of LWR on mRNA expression of hippocampal BDNF and TrkB.

	Group		
	Sed	No-loal	Load
**Body weight(g) **	390.2 ± 5.7	360.2 ± 5.6*	366.8 ± 5.3*
**Muscle mass and CS activity**			
Relative soleus wet mass to BW (mg/100g)	45.8 ± 0.8	49.2 ± 0.8*	47.9 ± 0.6
Relative plantaris wet mass to BW (mg/100g)	90.5 ± 0.8	95.9 ± 2.8*	94.9 ± 1.8
**Stress-related factors**			
Relative adrenals Wt to to BW (mg/100g)	14.9 ± 0.8	17.6 ± 1.0	18.2 ± 1.2
Relative thymus Wt to to BW (mg/100g)	145.4 ± 3.9	134.5 ± 8.8	138.5 ± 8.5

All data are presented as the mean ± SEM. # *p* < 0.01, * *p* < 0.05, compared to the sedentary group after ANOVA with Fisher’s PLSD post hoc test.

BW, body weight; CS, citrate synthase; Wt, weight.

**Table 2:** Effects of LWR on exercise performance. Fig.1. Effects of LWR on mRNA expression of hippocampal BDNF

	Group	
	Sed	No-loal
Wheel running performance		
Average running distance (m)	645.8 ± 110.5	621.9 ± 122.2
Average running time (min)	18.9 ± 3.2	16.9 ± 3.6
Average running velocity (m/min)	34.0 ± 0.7	36.6 ± 1.6
Average daily work levels (N*m/kg b.w/day)	28.4 ± 3.7	670.4 ± 94.6***

Average daily running distance, time, velocity and work levels are shown for rats in the No-load and Load groups. All data are presentedas the mean ± SEM. Significant difference compared with the Noloadgroup after Student’s t-test: *** *p* < 0.001

### Effect of LWR on skeletal muscle mass and CS activity

After completion of exercise, hind limb skeletal muscle masses (soleus and plantaris) were analyzed to determine the effect of wheel running. Relative soleus muscle wet masses in the No-load group were higher than those in the Sed group (P < 0.05; Table. 1). However, relative plantaris muscle wet masses had not changed compared to those in the Sed group. The soleus muscle CS activity in the Noload group was significantly higher than that in the Sed group (P < 0.05; Table 1). 

### Effect of LWR on stress-related factors

The adrenal and thymus weights were measured to investigate the possible roles of stress in LWR on hippocampal BDNF signaling. Our results revealed no significant changes in the relative weights of the adrenals and thymus among the three groups, which indicates the low negative stress effects of our resistance exercise model (Table 1). 

### LWR increased the BDNF mRNA and relationships between average work levels

**Fig. 1.**
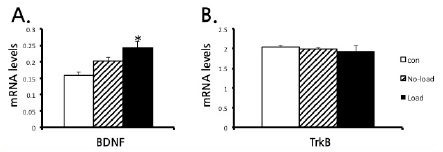
Effects of LWR on mRNA expression of hippocampal BDNF and TrkB. (A) BDNF mRNA increased only in Load groups. The expression of BDNF increased in the Load but not the No-load group. (B) Both No-load and Load groups was not changed the TrkB mRNA. All data are presented as the mean ± SEM. * *p* < 0.05, compared to sedentary after an ANOVA with Fisher’s PLSD post hoc test.

Analysis of quantitative real-time PCR data showed that BDNF mRNA expression in the Load group was significantly higher than that in the Sed group (P < 0.05; Fig. 1A). However, TrkB expression did not change in both the No-load and Load groups (Fig. 1B). 

### LWR increased the BDNF mRNA levels and relationships between average work levels

**Fig. 2.**
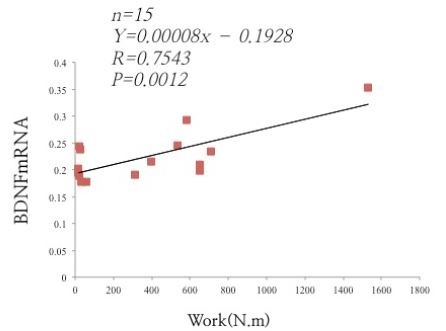
Effects of LWR on BDNF mRNA expression and relationships between average work levels. There is a significant correlation between the average work and hippocampal BDNF mRNA levels (Pearson’s correlation, r = 0.75, P < 0.001). Each point represents an individual rat.

A correlation analysis was performed between BDNF mRNA levels and work levels in individual animals. We found that BDNF mRNA levels were positively correlated with average work levels (r = 0.75, P < 0.001; Fig. 2). 

## DISCUSSION

This is the first study testing the hypothesis that acute additional resistance on a running wheel affects hippocampal BDNF signaling and neuronal plasticity. Here, we demonstrated that voluntary LWR, which causes functional adaptation in fast muscle, results in enhanced BDNF gene expression. LWR allows for the addition of a given load on a voluntary running wheel and is a useful exercise model for increasing the workload. LWR exercise elicited muscle hypertrophy and enhanced activity levels of the oxidative enzyme CS, probably due to the relatively high intensity and short duration of the exercise. In accordance with a previous study, which showed that voluntary wheel exercise increases hippocampal BDNF levels ^9^, our results show that LWR, which increased energy expenditure with load but did not increase running distance, not only replicates the effects of No-load on BDNF expression. This finding indicates the beneficial roles of voluntary resistance exercise on both muscular and hippocampal functional adaptations. 

In the present study, as expected, the performance of 1-week of LWR elicited distinct activity patterns that were affected by the application of a load equal to 30% of body mass. Compared to the No-load group, the average daily running distance, time, and velocity did not change with the resistance in the Load group. However, the work level of the Load group was 23.6 times higher than that in the No-load group. This is supported by the fact that the work levels accomplished could still be higher with Load compared to No-load ^15^^,^ ^16^^,^ ^24^, even if the average running distances decreased with resistance ^14^^,^ ^16^. These higher work levels with LWR would result in distinct muscular recruitment and adaptations across the muscles in both exercise groups. These results are consistent with our previous study, which found that CS activity was increased only in the LWR group ^17^^,^ ^18^. Moreover, the No-load group showed more pronounced effects on the slow-twitch soleus muscle ^25^, while the LWR elicited a greater effect on fast-twitch plantaris hypertrophy ^14^^,^ ^15^^,^ ^26^. Taken together, LWR as a voluntary exercise model with strength dependency is clearly different from the traditional voluntary wheel with distance precedency, and is an appropriate exercise for inducing physiological adaptation. 

Our results show that LWR resulted in no changes in plasma corticosterone levels and adrenal and thymus weights, indicating that LWR condision does not produce any of negative stress effects leading to general adaptation syndrome (GAS), thereby providing substantial beneficial effects on hippocampal BDNF signaling. Thus, LWR may indeed be an effective exercise model suitable for stimulating hippocampal function. 

The current study demonstrated up-regulated BDNF gene expression in the Load group. These results are in accordance with previous studies that showed that hippocampal BDNF mRNA expression was rapidly influenced by voluntary wheel running, and that BDNF Exon I-II mRNA expression levels were significantly up-regulated after 6 h of running and persisted after 12 h of voluntary running ^27^. BDNF plays an important role in hippocampal plasticity, and is thought to be a key molecule mediating the benefits of exercise on cognitive function ^7^^,^ ^28^^-^^30^. The importance of BDNF-TrkB signaling in exercise-enhanced cognitive function and adult hippocampal neurogenesis has been demonstrated since intra-hippocampal injection of the TrkB antibody abolished the beneficial effects of exercise on a hippocampus-dependent spatial learning task ^13^^,^ ^31^. 

Evidence has already shown that BDNF also regulates synaptic plasticity and several molecules such as PKC, MAPK ^32^^,^ Syn-I and NMDA-R ^7^. Moreover, exercise primes the molecular mechanisms responsible for synaptic gene encoding, resulting in a lowered threshold for the acquisition and facilitation of synaptic plasticity ^3^^,^ ^33^. BDNF also regulates synaptic plasticity-related molecules such as intracellular kinase signaling, mitogen-activated protein kinase (MAPK) and protein kinase C (PKC) ^32^, protein kinase A (PKA), cAMP response element-binding protein (CREB) ^34^, synaptic vesicle trafficking molecules (synapsin-I), and N-methyl-D-aspartate receptor (NMDA-R) ^35^. That LWR differentially affects the molecules mentioned above may further suggest that it also contributes to exercise-enhanced synaptic plasticity and neurotransmitter-trafficked processes via BDNF signaling. 

In the final set of findings, we confirmed that there was a significant correlation between average work level and BDNF mRNA expression (r=0.75). In short-term exercise (2–7 nights), there was a positive correlation between running distance and BDNF mRNA expression in the hippocampus ^9^. In contrast, after 40 days of wheel running, the correlation between hippocampal BDNF protein expression and running distance in mice no longer exists ^36^. Thus, the higher hippocampal BDNF mRNA expression may simply be due to the greater work levels after 1 week, implying that work levels are more important than running distance for acute exercise-enhanced hippocampal BDNF signaling. 

Collectively, this study demonstrates for the first time that acute 1 week of voluntary wheel running with resistance loading, which induces both muscle hypertrophy and oxidative enzyme hyperactivity, increased the gene expression of hippocampal BDNF signaling without causing a negative stress effect. This exercise resulted in a low running distance but enhanced energy expenditure, suggesting that “less is more” with respect to the effective running distance leading to brain plasticity. It is thus tempting to implicate that even short-distance running could have comparable beneficial effects as long-distance running on the hippocampal plasticity, if a proper resistance load is applied. Further studies to identify the specific mechanism underlying the strengthening exercise conditions inducing hippocampal plasticity are crucial to better understand the effects of strength exercise on hippocampus plasticity. 

## References

[1] Carek PJ, Laibstain SE, Carek SM. (2011). Exercise for the treatment of depression and anxiety. *Int J Psychiatry Med*.

[2] Al-Jarrah MD, Jamous M. (2011). Effect of endurance exercise training on the expression of GFAP, S100B, and NSE in the striatum of chronic/progressive mouse model of Parkinson's disease. *NeuroRehabilitation*.

[3] van Praag H, Christie BR, Sejnowski TJ, Gage FH. (1999). Running enhances neurogenesis, learning, and long-term potentiation in mice. *Proc Natl Acad Sci U S A*.

[4] Pencea V, Bingaman KD, Wiegand SJ, Luskin MB. (2001). Infusion of brain-derived neurotrophic factor into the lateral ventricle of the adult rat leads to new neurons in the parenchyma of the striatum, septum, thalamus, and hypothalamus. *J Neurosci*.

[5] Benraiss A, Chmielnicki E, Lerner K, Roh D, Goldman SA. (2001). Adenoviral brain-derived neurotrophic factor induces both neostriatal and olfactory neuronal recruitment from endogenous progenitor cells in the adult forebrain. *J Neurosci*.

[6] Lee J, Duan W, Mattson MP. (2002). Evidence that brain-derived neurotrophic factor is required for basal neurogenesis and mediates, in part, the enhancement of neurogenesis by dietary restriction in the hippocampus of adult mice. *J Neurochem*.

[7] Vaynman S, Ying Z, Gomez-Pinilla F. (2004). Hippocampal BDNF mediates the efficacy of exercise on synaptic plasticity and cognition. *Eur J Neurosci*.

[8] Poo MM. (2001). Neurotrophins as synaptic modulators. *Nat Rev Neurosci*.

[9] Neeper SA, Gomez-Pinilla F, Choi J, Cotman C. (1995). Exercise and brain neurotrophins. *Nature*.

[10] Soya H, Nakamura T, Deocaris CC, Kimpara A, Iimura M, Fujikawa T (2007). BDNF induction with mild exercise in the rat hippocampus. *Biochem Biophys Res Commun*.

[11] Barbacid M. (1994). The Trk family of neurotrophin receptors. *J Neurobiol*.

[12] Lindvall O, Kokaia Z, Bengzon J, Elmer E, Kokaia M. (1994). Neurotrophins and brain insults. *Trends Neurosci*.

[13] Li Y, Luikart BW, Birnbaum S, Chen J, Kwon CH, Kernie SG (2008). TrkB regulates hippocampal neurogenesis and governs sensitivity to antidepressive treatment. *Neuron*.

[14] Ishihara A, Roy RR, Ohira Y, Ibata Y, Edgerton VR. (1998). Hypertrophy of rat plantaris muscle fibers after voluntary running with increasing loads. *J Appl Physiol*.

[15] Legerlotz K, Elliott B, Guillemin B, Smith HK. (2008). Voluntary resistance running wheel activity pattern and skeletal muscle growth in rats. *Exp Physiol*.

[16] Call JA, McKeehen JN, Novotny SA, Lowe DA. (2010). Progressive resistance voluntary wheel running in the mdx mouse. *Muscle Nerve*.

[17] Lee MC, Okamoto M, Liu YF, Inoue K, Matsui T, Nogami H (2012). Voluntary resistance running with short distance enhances spatial memory related to hippocampal BDNF signaling. *J Appl Physiol (1985)*.

[18] Lee MC, Inoue K, Okamoto M, Liu YF, Matsui T, Yook JS (2013). Voluntary resistance running induces increased hippocampal neurogenesis in rats comparable to load-free running. *Neurosci Lett*.

[19] Lee MC, Rakwal R, Shibato J, Inoue K, Chang H, Soya H. (2014). DNA microarray-based analysis of voluntary resistance wheel running reveals novel transcriptome leading robust hippocampal plasticity. *Physiol Rep*.

[20] Kasuga N, Yamashita S, Ogasawara H, Suzuki H, Tsuzimoto H, Ishihara A. (1999). Various in running pattern and skeletal muscle adaptations in voluntary running rats at different load. *JJpn J Phys Fit Sport*.

[21] Lee MC, Okamoto M, Liu YF, Inoue K, Matsui T, Nogami H (2012). Voluntary resistance running with short distance enhances spatial memory related to hippocampal BDNF signaling. *J Appl Physiol*.

[22] Chomczynski P, Sacchi N. (1987). Single-step method of RNA isolation by acid guanidinium thiocyanate-phenol-chloroform extraction. *Anal Biochem*.

[23] Rozen S, Skaletsky H. (2000). Primer3 on the WWW for general users and for biologist programmers. *Methods Mol Biol*.

[24] Konhilas JP, Widegren U, Allen DL, Paul AC, Cleary A, Leinwand LA. (2005). Loaded wheel running and muscle adaptation in the mouse. *Am J Physiol Heart Circ Physiol*.

[25] Rodnick KJ, Reaven GM, Haskell WL, Sims CR, Mondon CE. (1989). Variations in running activity and enzymatic adaptations in voluntary running rats. *J Appl Physiol*.

[26] Ishihara A, Hirofuji C, Nakatani T, Itoh K, Itoh M, Katsuta S. (2002). Effects of running exercise with increasing loads on tibialis anterior muscle fibres in mice. *Exp Physiol*.

[27] Oliff HS, Berchtold NC, Isackson P, Cotman CW. (1998). Exercise-induced regulation of brain-derived neurotrophic factor (BDNF) transcripts in the rat hippocampus. *Brain Res Mol Brain Res*.

[28] Cotman CW, Berchtold NC. (2002). Exercise: a behavioral intervention to enhance brain health and plasticity. *Trends Neurosci*.

[29] Cotman CW, Berchtold NC. (2007). Physical activity and the maintenance of cognition: learning from animal models. *Alzheimers Dement*.

[30] Liu YF, Chen HI, Wu CL, Kuo YM, Yu L, Huang AM (2009). Differential effects of treadmill running and wheel running on spatial or aversive learning and memory: roles of amygdalar brain-derived neurotrophic factor and synaptotagmin I. *J Physiol*.

[31] Vaynman SS, Ying Z, Yin D, Gomez-Pinilla F. (2006). Exercise differentially regulates synaptic proteins associated to the function of BDNF. *Brain Res*.

[32] Gottschalk WA, Jiang H, Tartaglia N, Feng L, Figurov A, Lu B. (1999). Signaling mechanisms mediating BDNF modulation of synaptic plasticity in the hippocampus. *Learn Mem*.

[33] Farmer J, Zhao X, van Praag H, Wodtke K, Gage FH, Christie BR. (2004). Effects of voluntary exercise on synaptic plasticity and gene expression in the dentate gyrus of adult male Sprague-Dawley rats in vivo. *Neuroscience*.

[34] Vaynman S, Ying Z, Gomez-Pinilla F. (2003). Interplay between brain-derived neurotrophic factor and signal transduction modulators in the regulation of the effects of exercise on synaptic-plasticity. *Neuroscience*.

[35] Kitamura T, Mishina M, Sugiyama H. (2003). Enhancement of neurogenesis by running wheel exercises is suppressed in mice lacking NMDA receptor epsilon 1 subunit. *Neurosci Res*.

[36] Rhodes JS, van Praag H, Jeffrey S, Girard I, Mitchell GS, Garland T (2003). Exercise increases hippocampal neurogenesis to high levels but does not improve spatial learning in mice bred for increased voluntary wheel running. *Behav Neurosci*.

